# A Pharmacy-Based eHealth Intervention Promoting Correct Use of Medication in Patients With Asthma and COPD: Nonrandomized Pre-Post Study

**DOI:** 10.2196/32396

**Published:** 2022-06-08

**Authors:** Kyma Schnoor, Anke Versluis, Robbert Bakema, Sanne van Luenen, Marcel J Kooij, J Maurik van den Heuvel, Martina Teichert, Persijn J Honkoop, Job F M van Boven, Niels H Chavannes, Jiska J Aardoom

**Affiliations:** 1 Department of Public Health and Primary Care Leiden University Medical Center Leiden Netherlands; 2 National eHealth Living Lab Leiden University Medical Center Leiden Netherlands; 3 Nederlandse Service Apotheek Beheer 's Hertogenbosch Netherlands; 4 Department of Clinical Psychology Leiden University Leiden Netherlands; 5 Service Pharmacy Koning Amsterdam Netherlands; 6 Department of Respiratory Medicine Amsterdam University Medical Center University of Amsterdam Amsterdam Netherlands; 7 Department of Clinical Pharmacy and Toxicology Leiden University Medical Center Leiden Netherlands; 8 Department of Clinical Pharmacy and Pharmacology Groningen Research Institute for Asthma and COPD University Medical Center Groningen, University of Groningen Groningen Netherlands

**Keywords:** asthma, COPD, medication adherence, exacerbations, pharmacy, eHealth

## Abstract

**Background:**

Asthma and chronic obstructive pulmonary disease (COPD) affect millions of people worldwide. While medication can control and improve disease symptoms, incorrect use of medication is a common problem. The eHealth intervention SARA (Service Apothecary Respiratory Advice) aims to improve participants’ correct use of inhalation medication by providing information and as-needed tailored follow-up support by a pharmacist.

**Objective:**

The primary aim of this study was to investigate the effect of SARA on exacerbation rates in participants with asthma and COPD. Secondary aims were to investigate its effects in terms of adherence to maintenance medication and antimycotic treatment.

**Methods:**

In this nonrandomized pre-post study, medication dispensing data from 382 Dutch community pharmacies were included. Exacerbation rates were assessed with dispensed short-course oral corticosteroids. Medication adherence between new and chronic users was assessed by calculating the proportion of days covered from dispensed inhalation maintenance medication. Antimycotic treatment was investigated from dispensed oral antimycotics in participants who were also dispensed inhaled corticosteroids (ICS). Outcomes were assessed 1 year before and 1 year after implementation of SARA and were compared between SARA participants and control participants. More specifically, for exacerbation rates and medication adherence, a difference score was calculated (ie, 1 year after SARA minus 1 year before SARA) and was subsequently compared between the study groups with independent-samples *t* tests. For antimycotics, the relative number of participants who were dispensed antimycotics was calculated and subsequently analyzed with a mixed-effects logistic regression.

**Results:**

The study population comprised 9452 participants, of whom 2400 (25.39%) were SARA participants. The mean age of the population was 60.8 (15.0) years, and approximately two-thirds (n=5677, 60.06%) were female. The results showed an increase in mean exacerbation rates over time for both study groups (SARA: 0.05; control: 0.15). However, this increase in exacerbation rates was significantly lower for SARA participants (*t*_9450_=3.10, 95% CI 0.04-0.16; *P=*.002; Cohen *d*=0.06). Chronic users of inhalation medication in both study groups showed an increase in mean medication adherence over time (SARA: 6.73; control: 4.48); however, this increase was significantly higher for SARA participants (*t*_5886_=–2.74, 95% CI –3.86 to –0.84; *P*=.01; Cohen *d*=–0.07). Among new users of inhalation medication, results showed no significant difference in medication adherence between SARA and control participants in the year after implementation of SARA (*t*_1434_=–1.85, 95% CI –5.60 to 0.16; *P*=.06; Cohen *d*=–0.10). Among ICS users, no significant differences between the study groups were found over time in terms of the proportion of participants who were dispensed antimycotics (*t*_5654_=0.29, 95% CI –0.40 to 0.54; *P*=.76; Cohen *d*=0).

**Conclusions:**

This study provides preliminary evidence that the SARA eHealth intervention might have the potential to decrease exacerbation rates and improve medication adherence among patients with asthma and COPD.

## Introduction

Asthma and chronic obstructive pulmonary disease (COPD) are chronic respiratory diseases that affect millions of people worldwide [1,2]. Asthma and COPD place a significant health burden on patients and an economic burden on society [3-5]. Medication cannot cure these diseases but can reduce disease symptoms and improve control, which, in turn, can positively affect patients’ quality of life [6-9]. Unfortunately, nonadherence to maintenance medication is common in patients with asthma and COPD. Indeed, adherence rates have been found to vary from 22% to 78% [7]. Nonadherence can have detrimental effects on clinical outcomes for individuals with asthma and COPD. Notably, it could negatively affect lung function, disease control, exacerbation rate, health-related quality of life, and work productivity [6,7,10]. In addition, nonadherence has been associated with higher health care use and costs [6,7].

Factors related to nonadherence to inhaled medication are multifaceted and can include intentional nonadherence (eg, concerns about side effects and complexity of medication regime) and unintentional nonadherence (eg, experiencing difficulties with how or when to use medication or lacking skills to use inhaler devices) [7,9,11-15]. Regarding incorrect use of the inhalers, Lavorini et al [12] systematically investigated the use of dry powder inhalers by patients with asthma or COPD. The results showed that between 4% and 94% of the patients did not use their inhalers correctly, with exact rates depending on the type of inhaler and the assessment method used. As such, these patients need additional care to support correct medication usage, and effective intervention strategies are required.

A variety of strategies have been investigated that aim to tackle the problem of nonadherence. Training and education on correct inhaler technique are considered crucial in combating nonadherence [9] and in effectively managing one’s asthma or COPD [16]. A Cochrane systematic review demonstrated the efficacy of interventions intended to improve adherence to inhaled corticosteroids (ICS) among patients with asthma [17]. Adherence education, electronic trackers or reminders, and simplified regimens were found to improve adherence by 20%, 19%, and 4%, respectively [17]. Recently, a meta-analysis by Jeminiwa et al [18] also showed a positive effect of eHealth strategies on improving adherence to ICS among people with asthma. However, according to the Cochrane systematic review, clinical outcomes are often not improved with those interventions [17].

In the Netherlands, the eHealth intervention SARA (Service Apothecary Respiratory Advice; in Dutch, Service Apotheek Raad en Advies) was developed to promote correct use of inhalation medication for patients with asthma and COPD. The goal of this self-management intervention is to reduce the burden of lung disease and reduce exacerbations by stimulating correct use and adherence of inhaler medication in patients with asthma and COPD. SARA combines several interventions’ components, including education, self-management strategies, and as-needed follow-up care by a pharmacist.

This study aimed to investigate the effectiveness of SARA in participants with asthma and COPD by comparing pharmacy dispensing data over time, that is, before and after the implementation of SARA, as well as between SARA participants and a control group. The primary aim of this study was to investigate the effect of SARA on exacerbation rates. The secondary aims were to investigate the effect of SARA on medication adherence and antimycotic treatment.

## Methods

### The SARA eHealth Intervention

The SARA eHealth intervention was developed by the Service Pharmacy organization. The Service Pharmacy organization supports independent but affiliated community pharmacies (ie, Service Pharmacies) in their day-to-day business operations to provide high-quality pharmaceutical care and provide offline and online communication. The Service Pharmacy organization developed SARA to support and prepare pharmacies for the second dispensing of inhalation medication. Pilot studies were then conducted with SARA and its corresponding questionnaire. Relevant input on how to improve the intervention was gathered by conducting two focus group interviews with pharmacists as well as patients with asthma and COPD, gaining insight into their needs and preferences. Their input was used to improve the intervention where possible.

SARA aims to improve the correct use of inhalation medication by providing information and supporting knowledge about this type of medication. SARA is an online platform that contains the following: (1) comprehensive information about inhalation medication, its usage, and side effects; (2) inhalation instruction videos; (3) informational videos about asthma and COPD; (4) a pollen forecast; and (5) a questionnaire that is emailed to individuals on the 15th day after starting SARA. A 7-item questionnaire was developed by the Service Pharmacy organization, assessing patients’ inhalation medication usage and related experiences, concerns and doubts, difficulties, and side effects (Multimedia Appendix 1). The questionnaire was based on the national Dutch guideline for pharmaceutical patient consultation, specifically regarding the second dispensing of inhalation medication, which was in development at the time [19]. This consultation guideline aims to support the community pharmacist in providing patient-centered care during pharmaceutical consultations provided by the pharmacist to the patient. The seven drafted questions were discussed in a focus group with pharmacists, and the feedback was subsequently used to improve the questionnaire to maximize its reliability. The outcomes of the questionnaires are automatically forwarded to the corresponding pharmacy. Next, the pharmacist can provide as-needed follow-up care in case any important issues are encountered, such as experiencing one or more severe side effects. The type and intensity of follow-up care can be tailored to the identified patient needs and preferences and the pharmacist’s resources. Pharmacists are trained to identify patients’ individual needs before delivering additional support, especially because SARA identifies those with extra needs. The follow-up care can entail additional detailed inhalation instructions or training (eg, when a patient experiences difficulties inhaling), providing additional information on how to properly use the medication (eg, when a patient reports not knowing when to take the medication or whether one can use the medication in combination with other medication), or providing additional information on the importance of taking the medication and its effects (eg, when a patient reports not having taken the medication because of doubts about whether it will work). The follow-up care can be offered through extra pharmacy visits, extra house visits, telephone calls, or digital communication tools, such as chats.

### Design

This study entailed a nonrandomized pre-post study design. Pharmacy dispensing data were used to compare patient-level medication dispensing data over time (ie, the year before versus after implementation of SARA, hereafter often referred to as “over time”) and between groups (ie, SARA versus control participants).

### Ethical Considerations

No ethics approval was applied for because this study was declared to not fall within the scope of the Dutch Medical Research Involving Human Subjects Act by the Medical Ethics Committee (MEC) of the Leiden University Medical Center (MEC No. G20.030).

### Participant Flow

From the beginning of 2017 onward, SARA has been implemented in approximately 400 Service Pharmacies in the Netherlands. Not all Service Pharmacies participated in SARA. Some pharmacies could not participate in SARA because of conflicting software programs, among other reasons. Other pharmacies declined to participate in SARA due to personnel problems, thereby resulting in not having the resources to implement a different and new way of working.

In the participating pharmacies, individuals were offered SARA during a pharmacy visit when collecting inhalation medication for their asthma, COPD, bronchitis, or another indication. More specifically, individuals were offered SARA when they were dispensed medication for obstructive airway disease within the R03 class of drug, according to the use of the Anatomical Therapeutic Chemical (ATC) classification as developed by the World Health Organization (WHO) [20]. The trigger for pharmacists to invite a patient to participate in SARA was dispensing of an R03 class of drug. However, pharmacists could choose not to offer SARA to patients if they considered them ineligible for participation in SARA, for example, those living in a nursing home or those with very limited digital literacy levels. When interested in SARA, participants were subsequently enrolled in the intervention. Otherwise, they were asked to indicate whether they were not interested in SARA at that specific point in time or would never be interested. Patients’ choices were registered by the pharmacists in the pharmacy dispensing database, as well as the date their choices were registered, from here on referred to as the “registration date.” If patients wanted to participate, they were enrolled by their pharmacist in the SARA program, after which they were sent a registration confirmation link and were able to start the program accordingly. The process of registering patients’ choices in the database was sometimes delayed in daily practice, with pharmacists conducting the formal registration in the pharmacy dispensing database a while after the actual dispensing. Patients who were interested and subsequently agreed to participate in SARA were categorized as SARA participants. Those who were not interested were categorized as control participants. Additionally, patients who collected their inhalation medication and who were never offered SARA were categorized as control participants as well.

**Figure 1:**
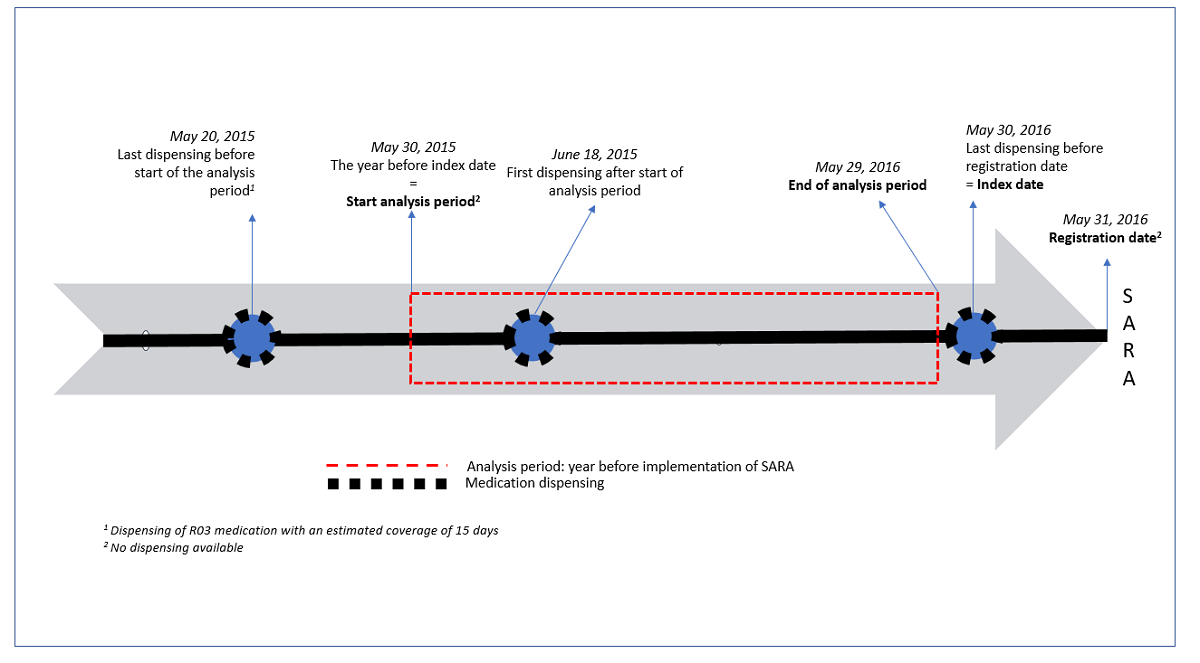
Operationalized analysis period for the year before the implementation of SARA. Step 1: the index date (ie, May 30, 2016) was used to calculate the specific period of analysis (ie, the day before the index date = the end of the analysis period before the implementation of SARA). Step 2: medication adherence scores were calculated based on the proportion of days covered with the "at least one" method. SARA: Service Apothecary Respiratory Advice; in Dutch, Service Apotheek Raad en Advies.

The index date was calculated using one of the following two options: (1) if there was an R03-medication dispensing available on the registration date, the registration date was defined as the study index date, or (2) if there was no R03-medication dispensing available on the registration date, the last dispensing date before the registration date was defined as the study index date. Subsequently, the index date was used to calculate the specific period of analysis (ie, the year before as well as the year after implementation of SARA) for each participant. More specifically, the index date was coded as the starting date of the year of analysis after the implementation of SARA. The exact year of analysis before implementation of SARA was coded as the year before the index date, not including the index date itself. Figure 1 presents an example of the index date calculation using option 2, in which case the registration date of the participant was May 31, 2016. As no medication dispensing was available for this date, the last dispensing date before the registration date (ie, May 30, 2016) was taken as the index date. Subsequently, May 30, 2016, was set as the starting date of the year after implementation, whereas the year before implementation of SARA would cover the period up to and including May 29, 2016.

### Study Population

Medication dispensing data from January 2015 to September 2020, from 382 Service Pharmacies located in different regions of the Netherlands, were obtained by information and communications technology service provider NControl. Patients’ data in the NControl database are pseudonymized, meaning that their data cannot be directly connected to the natural person (ie, data subject) to whom they belong without the use of additional information, which is kept separately, according to Article 4(5) of the General Data Protection Regulation [21]. NControl provided a selection of this pseudonymized data to the main researchers of the Leiden University Medical Center, including data on patient demographics (ie, year of birth and gender), disease indication (ie, asthma, COPD, bronchitis, or other), the name of the Service Pharmacy, and medication dispensing records with detailed information on the type of the dispensed medication, ATC codes, corresponding dispensing date, amount dispensed, estimated covering days, and prescribed daily dosage. These data were not attributable to specific data subjects*;* these subjects were represented by personal identifier numbers that could not be used to directly identify a natural person (ie, data subject).

The study population consisted of individuals collecting R03 medication at one of the included 382 Service Pharmacies. Eligibility criteria to be included in the analyses were as follows: (1) patients aged 18 years or older at the time of their first available dispensing date record, (2) patients registered as SARA or control participants (ie, no missing data on SARA participation status), and (3) the time between the index date and the most recent R03-medication dispensing was a maximum of 30 days. This third inclusion criterion was chosen because SARA was always offered during a participant’s pharmacy visit for collecting one’s R03 medication, and if the time between this dispensing date and the registration date was more than 30 days, we considered it as a potential source of bias. We then presumed that it indicated a significant delay in the pharmacists’ registration of SARA participation, which would result in uncertainty about what period to operationalize as “before implementation of SARA” and what period to operationalize as “after implementation of SARA.” The fourth eligibility criterion was that patients had to have a disease indication from the pharmacy for asthma or COPD, excluding patients with indications other than asthma or COPD. The fifth and final eligibility criterion was that patients had to have at least one medication dispensing record before starting the 2-year analysis period and at least one record after, in order to ensure complete and up-to-date dispensing data during the analysis period. Besides the five eligibility criteria mentioned above, additional outcome-specific eligibility criteria were in place for the secondary outcomes of medication adherence and antimycotic treatment (see the respective subsections in the Outcome Measures section).

### Outcome Measures

#### Exacerbation Rates

The primary outcome measure was the difference in exacerbation rates over time (ie, before versus after implementation of SARA) between SARA and control participants. The medication dispensing data of short-course prednisone and prednisolone, hereafter referred to as prednisone, were used to estimate exacerbation rates, as prednisone is prescribed to inhibit the inflammation of exacerbations. Prescriptions with ATC codes H02AB06 (prednisolone) and H02AB07 (prednisone) were used to estimate exacerbation rates. The medication dispensing records were categorized as exacerbations based on the Dutch College of General Practitioners’ guidelines for asthma and COPD [22,23], that is, in the case of a dispensing record reflecting a daily dosage of 30 or 40 mg of prednisone for a minimum of 5 days and a maximum of 14 days. The mean number of exacerbations in the year before and after implementation of SARA was summed into a mean total score of exacerbations for each of these analysis periods.

#### Medication Adherence

One of the secondary outcomes was the difference in medication adherence over time between SARA and control participants. In addition to the general eligibility criteria as mentioned in the Study Population section, another inclusion criterion was formulated for this outcome measure. Participants needed to have at least three dispensing records of R03 medication during the 2-year analysis period in order to exclude fully nonadherent participants and validate the method of calculating medication adherence. In this way, participants with early cessation were excluded from the calculation, and only patients who were pharmacologically treated were included in the analyses.

The WHO definition of adherence was used to operationalize medication adherence, that is, the extent to which a person’s behavior corresponds with the agreed-upon recommendations from a health care provider [15]. Studying medication adherence using medication dispensing records of pharmacies is a common method for assessing adherence [24]. Relevant groups of inhalation medication according to the WHO ATC classification included R03 medication, that is, medication for obstructive airway diseases [25]. All medication dispensings of the maintenance R03 medications represented by the following codes were included in the database: R03BA01, R03BA02, R03BA05, R03BA08, R03AK06, R03AK07, R03AK08, R03AK10, R03AK11, R03AL03, R03AL04, R03AL05, R03AL08, R03AL09, R03AC18, R03AC13, R03AC12, R03BB04, R03BB05, R03BB06, and R03BB07. These included ICS, long-acting beta agonists, long-acting muscarinic antagonists, and fixed-dose combinations. Nebulizers were excluded from the analyses.

Medication adherence was operationalized as the proportion of days covered (PDC). The PDC is the preferred method for calculating adherence at a population level and has been operationalized by the Pharmacy Quality Alliance [26]. In this study, the PDC was defined as the ratio of the number of days that a patient had medication available for at least one type of R03 medication during exactly 1 analysis year (ie, before and after the implementation of SARA, respectively) to the total number of days that the patient was dispensed the medication during that same period (ie, estimated covering days of the medication). Hence, the PDC reflected the proportion of days that the individual had at least one type of R03 medication available during the corresponding year of analysis.

More specifically, the “at least one” method was applied, which is a standardized method for measuring concurrent adherence to multiple related medications, in this case, the broad class of R03 medications. When the estimated coverage period of dispensed R03 medication did not precisely cover all 365 days of the 1-year analysis period, the data from the first available R03-medication dispensing record before or after the analysis period, respectively (ie, depending on whether it concerned the analysis period before or after implementation of SARA), was used to determine the coverage of days belonging to the analysis period. Two assumptions were made in this process: (1) participants would only come to collect R03 inhalation medication once they finished their previously collected medication; in this way, the stock was not taken into account, and (2) participants would fully adhere to the prescribed dosage from the dispensing date onward until the end of the prescribed covering days. The above-mentioned methods and flow of this calculation of the PDC is presented in Figure 1.

**Figure 2:**
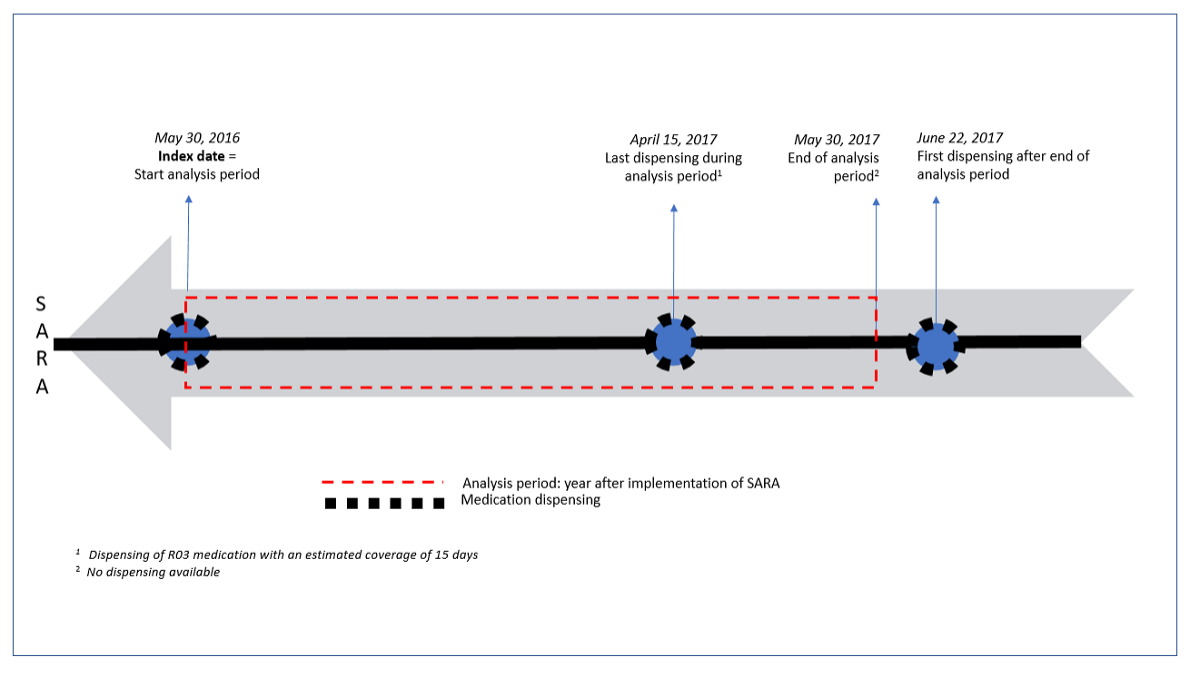
Operationalized analysis period for the year after the implementation of SARA. Step 1: the index date (ie, May 30, 2016) was used to calculate the specific period of analysis (ie, index date = the start of the analysis period after the implementation of SARA). Step 2: medication adherence scores were calculated based on the proportion of days covered with the "at least one" method. SARA: Service Apothecary Respiratory Advice; in Dutch, Service Apotheek Raad en Advies.

Looking at Figure 1, a patient’s analysis period before implementation of SARA started on May 30, 2015, but no medication dispensing was available for this date. The last dispensing before the start of this analysis period was on May 20, 2015, with an estimated coverage of 15 days, that is, the period of May 20 to June 3, 2015. The period from June 4, 2015, onward to the day before the next medication dispensing on June 18, 2015 (ie, the period from June 4 up to and including June 17, 2015), would be coded as “not covered.” Similarly, looking at Figure 2, for example, a patient’s analysis period after implementation of SARA ended on May 30_,_ 2017, and the last available dispensing record concerned a dispensing of R03 medication on April 15, 2017, with an estimated coverage of 15 days. This last dispensing thus covered the period from April 15 to 29, 2017. No records of dispensing data were available for the period from April 30 to May 30, 2017; hence, this period was coded as “not covered.” Medication adherence scores could range from 0 to 100, where 100 would reflect all 365 days of the analysis year being covered.

As it is commonly a cutoff point for good adherence, the PDC of 0.8 was used [26,27]. If it could not be determined whether or not a patient was covered by medication for a specific day of the year, a PDC could not be calculated; this would be considered a missing value. 

The analyses were performed separately for *new users* and *chronic users* of R03 medication because different behaviors were expected for these two groups [28]. New users refer to participants starting with inhalation medication, operationalized as zero R03 dispensing records in the year before the index date. Chronic users refer to those already using R03 medication, operationalized as having at least one R03 dispensing record in the year before the index date.

#### Antimycotic Treatment

Antimycotic treatment was operationalized as the difference over time in dispensed antimycotics between the SARA and control participants. The prevalence of oral candidiasis, potentially associated with ICS use, was estimated based on dispensing data of antimycotics in the subpopulation of participants who were dispensed ICS during the analysis period. Therefore, an additional inclusion criterion was formulated: participants needed to have at least one medication dispensing record of ICS (ie, ATC code R03BA01, R03BA02, R03BA05, or R03BA08) during the analysis period. If a participant was dispensed antimycotics (ie, ATC code J02AC01 [fluconazole], J02AC02 [itraconazole], A07AA02 [nystatin], A07AA07 [amphotericin B], or A07AC01 [miconazole]) during the analysis period, the outcome was coded as 1 (“yes”); if not, the outcome was coded as 0 (“no”). Next, the percentage of participants with an antimycotic dispensing was calculated per study condition and subsequently compared before and after the implementation of SARA.

### Statistical Analyses

The study population characteristics, per outcome measure, were summarized by descriptive statistics: means and SDs for continuous variables, and counts and percentages for dichotomous and categorical variables. Potential differences between SARA and control participants were analyzed using *t* tests for normally distributed continuous variables and chi-square tests for categorical variables.

Differences in the outcome measures of exacerbation rates and medication adherence were analyzed using independent *t* tests to examine potential differences between the two study groups over time. More specifically, difference scores were calculated per patient by subtracting the outcome scores (ie, exacerbation rates and PDC sores for the subpopulation of chronic users of inhalation medication) of the year before implementation of SARA and the scores in the year after. Additionally, for the subpopulation of new users of inhalation medication, an independent-samples *t* test was conducted to investigate differences in medication adherence in the year after implementation of SARA between SARA and control participants. The potential effects of covariates (ie, age and gender) were tested by means of analysis of covariance. The results of these analyses were only presented in the case of significant effects of covariates.

A mixed-effects logistic regression was conducted to analyze the change over time between the two study groups regarding the relative number of patients who were dispensed antimycotics. In this analysis, an interaction term of time (ie, before and after the index date) and the study condition (ie, SARA vs control) was included to analyze the change over time across groups. The potential effects of covariates (ie, age and gender) were tested by adding those as interaction terms to the model. The results of these analyses were only presented in the case of significant effects of covariates.

All analyses were conducted in the total population consisting of both patients with asthma and those with COPD. For exploratory purposes, separate analyses for the subpopulations of patients with asthma and those with COPD were conducted. For all the analyses, a significance level of *P*≤.05 was used, and a Cohen *d* was calculated to measure effect sizes. All analyses were conducted in SPSS Statistics for Windows (version 25.0; IBM Corp).

## Results

### Study Population

**Figure 3:**
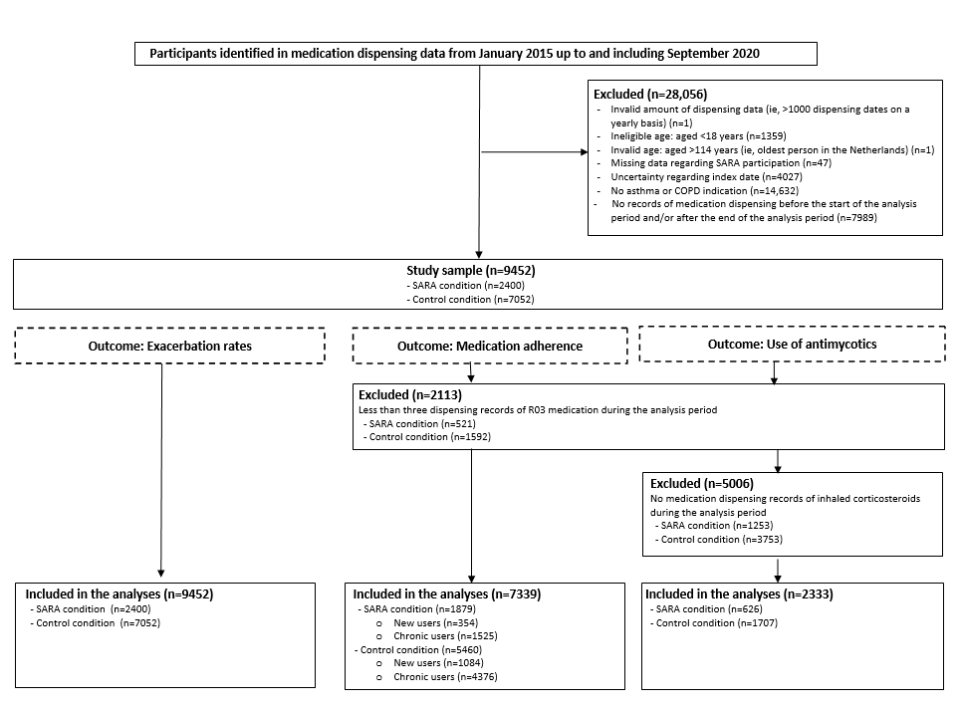
Flow of participants for the different outcome measures and corresponding analyses. SARA: Service Apothecary Respiratory Advice; in Dutch, Service Apotheek Raad en Advies.

The flow of included patients is presented in Figure 3. The total study population comprised of 9452 individuals with either asthma or COPD. Of those, 25.39% (n=2400) were enrolled in SARA, 25.73% (n=2432) indicated that they were not interested in using SARA, and 48.88% (n=4620) were not invited to participate or indicated that they did not want to start using SARA at that particular moment in time. As the inclusion criteria differed per outcome measure, the demographic characteristics are presented separately for each outcome measure (Table 1). Overall, the mean age of the study population was 60.8 (SD 15.0) years, and almost two-thirds of the study population were female. In all the different subpopulations, the mean age of patients using SARA was significantly lower than that of patients in the control group. In general, there was a significantly larger proportion of men in the control group as compared to the SARA group. Table S1 in Multimedia Appendix 2 shows the characteristics of the study samples separately per disease indication for asthma and COPD.

**Table 1:** Demographic characteristics of the study populations analyzed for the different outcome measures.

Outcome measure and characteristics						SARA^a^ participants (n=2400)			Control participants (n=7052)			Total population (N=9452)		*P* value^b^
**Exacerbation rate**														
	**Gender, n (%)**													
		Male			882 (36.75)			2851 (40.43)			3733 (39.49)			.002
		Female			1504 (62.67)			4173 (59.17)			5677 (60.06)			
		Unknown			14 (0.58)			28 (0.40)			42 (0.44)			
	Age (years), mean (SD)				57.7 (13.8)			61.9 (15.3)			60.8 (15.0)			<.001
**Medication adherence**														
	**Total group (SARA: n=1879; control: n=5460; total: n=7339)**													
		**Gender, n (%)**												
			Male	693 (36.88)			2200 (40.29)			2893 (39.42)			.01	
			Female	1175 (62.53)			3239 (59.32)			4414 (60.14)				
			Unknown	11 (0.58)			21 (0.38)			32 (0.44)				
		Age (years), mean (SD)			60.9 (13.4)			65.1 (14.5)			64.0 (14.4)			<.001
	**Subgroup: new users^c^ (SARA: n=354; control: n=1084; total: n=1438)**													
		**Gender, n (%)**												
			Male	128 (36.16)			420 (38.74)			548 (38.11)			.38	
			Female	225 (63.56)			658 (60.70)			883 (61.40)				
			Unknown	1 (0.28)			6 (0.55)			7 (0.49)				
		Age (years), mean (SD)			59.4 (14.2)			62.7 (16.5)			61.9 (16.0)			.002
	**Subgroup: chronic users^d^ (SARA: n=1525; control: n=4376; total: n=590)**													
		**Gender, n (%)**												
			Male	565 (37.05)			1780 (40.68)			2345 (39.74)			.02	
			Female	950 (62.29)			2581 (58.98)			3531 (59.84)				
			Unknown	10 (0.66)			15 (0.34)			25 (0.42)				
		Age (years), mean (SD)			61.3 (13.1)			65.7 (14.0)			64.6 (13.9)			.04
**Antimycotic treatment** **(SARA:** **n=626; control: n=1707; total: n=2333)**														
	**Gender, n (%)**													
		Male			196 (31.31)			612 (35.85)			808 (34.63)			.04
		Female			428 (68.37)			1090 (63.85)			1518 (65.07)			
		Unknown			2 (0.32)			5 (0.29)			7 (0.30)			
	Age (years), mean (SD)				55.1 (14.2)			59.0 (16.2)			58.0 (15.8)			<.001

^a^SARA: Service Apothecary Respiratory Advice; in Dutch, Service Apotheek Raad en Advies.

^b^*P* values represent comparisons between the SARA group and the control group; for characteristics with multiple subcategories (ie, gender), values for the group are reported in the top row of the group.

^c^New users are participants with zero R03 dispensing records in the year before the index date.

^d^Chronic users are participants with at least one R03 dispensing record in the year before the index date.

### Exacerbation Rates

In the year before the implementation of SARA, 63.00% (5955/9452) of the total study population had 0 exacerbations (range 0-12). In the year after the implementation of SARA, 56.00% (5293/9452) of the study population had 0 exacerbations (range 0-14). In both study groups, the mean rate of exacerbations was higher in the year after the implementation of SARA (SARA: mean 0.73; control: mean 0.82) than in the year before (SARA: mean 0.68; control: mean 0.67). Yet, as shown in Table 2, there was a significant difference between the SARA and control participants regarding the exacerbation rate over time, showing that the increase in exacerbations was significantly less in the SARA group (*P*=.002)*.* The results of the exploratory analyses are presented in Table S2 in Multimedia Appendix 2. In both participants with asthma and those with COPD, the mean exacerbation rate increased over time in both the SARA group (asthma: mean increase 0.07; COPD: mean increase 0.03) and the control group (asthma: mean increase 0.17; COPD: mean increase 0.12). As presented in Table S2 in Multimedia Appendix 2, among the asthma participants, the difference in exacerbation rates differed significantly between study groups (*P*=.003), indicating that SARA participants had a significantly lower increase in exacerbation rates over time in comparison to the control participants. No significant difference between the SARA and control participants was found in the COPD population regarding the change in exacerbation rate over time (Table S2 in Multimedia Appendix 2).

**Table 2:** Outcome results in terms of exacerbation rates.

Study group and periods^a^		Exacerbation rate, mean (SD)	Difference score^b^	Participants (N=9452), n (%)	*t* test (*df*)^c^	*P* value^c^	95% CI^c^	Cohen *d*^c^
**Control**								
	1 year before	0.67 (1.2)		7052 (74.61)	3.10 (9450)	.002	0.037-0.163	0.06
	1 year after	0.82 (1.3)	0.15	7052 (74.61)				
**SARA**								
	1 year before	0.68 (1.2)		2400 (25.39)				
	1 year after	0.73 (1.2)	0.05	2400 (25.39)				

^a^The study periods were 1 year before and 1 year after the implementation of SARA (Service Apothecary Respiratory Advice; in Dutch, Service Apotheek Raad en Advies).

^b^The difference score was calculated as the exacerbation rate the year after SARA minus the rate the year before SARA; values are only reported in the “1 year after” rows.

^c^Statistics comparing study groups are reported only in the top row of values.

### Medication Adherence

In both study groups, the mean PDC in the subpopulation of chronic users was higher in the year after compared to the year before implementation of SARA for both SARA participants (after: mean 77.26; before: mean 70.53) and control participants (after: mean 77.77; before: mean 73.29). However, there was a significant difference in change over time between the SARA and the control groups, showing that the increase in medication adherence was significantly higher in the SARA group (Table 3).

The exploratory results, repeating the analyses for the chronic user subgroup of participants with asthma and participants with COPD, are presented in Table S3 in Multimedia Appendix 2. For patients with asthma who were chronic users, there was an increase in medication adherence with no significant difference between the SARA and control participants. Gender was found to be a significant covariate for the patients with COPD who were chronic users. Splitting the analyses for men and women within this subpopulation showed that the increase in medication adherence for men was significantly higher for SARA participants than for control participants. For women, there was no significant difference between SARA and control participants over time in terms of medication adherence.

When comparing medication adherence in the year after implementation of SARA between the study groups for new users with COPD, this population showed significantly higher medication adherence in the SARA group as compared to the control group (Table S4 in Multimedia Appendix 2). No significant difference between the study groups was found in the subpopulation of new users with asthma.

**Table 3:** Outcome results in terms of medication adherence among the chronic user subpopulation.

Study group and periods^a^			PDC^b^, mean (SD)		Days covered, mean (SD)		Difference score^c^		Participants (n=5888),n (%)		*t* test (*df*)^d^		*P* value^d^		95% CI^d^		Cohen *d*^d^
**Control**																	
	1 year before	73.29 (28.3)		267.50 (103.4)				4368 (74.18)		–2.74 (5886)		.01		–3.856 to –0.839		–0.07	
	1 year after	77.77 (25.2)		283.86 (91.8)		4.48		4368 (74.18)									
**SARA**																	
	1 year before	70.53 (29.8)		257.45 (108.6)				1520 (25.82)									
	1 year after	77.26 (25.0)		282.01 (91.1)		6.73		1520 (25.82)									

^a^The study periods were 1 year before and 1 year after the implementation of SARA (Service Apothecary Respiratory Advice; in Dutch, Service Apotheek Raad en Advies).

^b^PDC: proportion of days covered.

^c^The difference score was calculated as the PDC 1 year after SARA minus 1 year before SARA; values are only reported in the “1 year after” rows.

^d^Statistics comparing study groups are reported only in the top row of values.

### Antimycotic Treatment

As shown in Table 4, the relative mean number of participants who had been dispensed antimycotics was higher after the implementation of SARA as compared to the year before for both SARA participants (6.4% vs 5.4%) and control participants (6.1% vs 4.7%). Results showed no significant differences in the relative number of participants who had been dispensed both ICS and antimycotics between the SARA and control groups (*P*=.82). Additionally, in the exploratory results, no significant differences were found with respect to antimycotic treatment over time between SARA and the control participants in the subgroups of participants with asthma and COPD (Table S5 in Multimedia Appendix 2).

**Table 4:** Results of the mixed-effects logistic regression regarding dispensed antimycotics among participants who were dispensed ICS.

Study group and periods^a^			Participants dispensed antimycotics, n (%)		Participants dispensed ICS^b^, n (%)		*t* test (*df*)^c^		*P* value^c^		95% CI^c^		Cohen *d*^c^
**Control (n=1707)**													
	1 year before	80 (4.69)		1707 (73.17)		0.23 (4662)		.82		–0.461 to 0.584		0	
	1 year after	104 (6.09)		1707 (73.17)									
**SARA (n=626)**													
	1 year before	34 (5.43)		626 (26.83)									
	1 year after	40 (6.39)		626 (26.83)									

^a^The study periods were 1 year before and 1 year after the implementation of SARA (Service Apothecary Respiratory Advice; in Dutch, Service Apotheek Raad en Advies).

^b^ICS: inhaled corticosteroids; percentages are based on total participants in both groups (n=2333).

^c^Statistics comparing study groups are reported only in the top row of values.

## Discussion

### Principal Findings

This study investigated the effectiveness of the pharmacy-based eHealth intervention SARA by comparing pharmacy dispensing data between SARA and control participants over time before and after the implementation of SARA. The results showed a smaller increase in exacerbation rates over time for SARA participants as compared to control participants. Furthermore, in the SARA group, chronic users of inhalation medication had a significantly larger increase in medication adherence over time as compared to control participants. Finally, no significant differences between the study groups were found with respect to antimycotic treatment over time.

Although the observational data do not entirely allow for causal conclusions, the significantly smaller increase in exacerbation rates over time among SARA participants may suggest a beneficial effect of SARA. Earlier clinical intervention studies comprising a behavioral intervention and integrated disease management program have also found positive effects on exacerbation rates among asthma participants [29,30]. Yet, SARA has the potential to help control exacerbations in a less invasive and less time-consuming way; this is potentially apparent in reduced material and immaterial costs, such as less time spent conducting follow-ups by pharmacists.

The results regarding medication adherence showed that chronic users of inhalation medication in the SARA group had a significantly higher increase in medication adherence as compared to control participants. This finding aligns with a previous meta-analysis examining eHealth strategies to improve medication adherence in ICS users [18]. However, it is essential to note that the mean medication adherence was lower for SARA participants than control participants, both before and after the implementation of SARA. A potential explanation is selection bias. Patients with more severe symptoms may have been more likely to be invited to participate in the SARA intervention by the pharmacists because they may visit the pharmacy more often, and patients with more severe symptoms typically show lower medication adherence [10]. On the other hand, patients with more severe symptoms may simply have been more interested in participating in the SARA intervention considering their higher disease burden, which may have, in turn, biased the results. The finding that new users of inhalation medication generally had lower medication adherence scores than chronic users emphasizes the importance of analyzing those two patient groups separately, as they appear to have different adherence patterns.

An interesting difference between men and women was found in the analysis of patients with COPD who were chronic users of inhalation medication. The results suggested that men within this subpopulation benefitted more from SARA (ie, increased medication adherence in comparison to controls) than women (ie, no differences between SARA and control participants). Little research is available on gender-associated differences in response to self-management interventions. A narrative review did discuss some evidence that women have more trouble with using inhalation medication correctly [31]. Furthermore, a systematic review discussed mixed results regarding gender-associated differences in response to pulmonary rehabilitation [32]. Thus, there appears to be some evidence of gender-associated differences that could explain our finding; however, more research is needed to investigate individual differences of patients regarding adherence based on their characteristics, beliefs, and attitudes to adherence.

With respect to antimycotic treatment for oral candidiasis in a subpopulation of ICS users, no difference was found between the study groups over time. These results should be interpreted carefully because the included sample was small, possibly limiting the power to detect statistical significance. To our knowledge, this was the first study that analyzed the effect of an eHealth intervention for patients with asthma and COPD on antimycotic treatment. The exploratory analyses showed a more favorable course of exacerbation rates over time for SARA versus control participants in the subpopulation of patients with asthma. This effect was not found in the subpopulation of patients with COPD. Our results are in line with previous research investigating a clinic-based intervention aiming to improve inhaler techniques, which only showed a positive effect in patients with asthma but not in patients with COPD [33]. It might be that patients with asthma benefit more from the educational intervention elements than patients with COPD. Alternatively, it might be due to more difficulties in managing COPD symptoms as the disease progresses, or the fact that COPD often results from smoking and that smoking cessation is quite challenging.

Furthermore, exploratory results showed that new users of inhalation medication had higher medication adherence in the year after SARA implementation among SARA participants as compared to control participants, but only in the subpopulation of patients with COPD and not in patients with asthma. In addition, patients with COPD generally had higher medication adherence than patients with asthma. This is in line with literature showing that patients with COPD generally have better adherence rates than patients with asthma, and there are multiple explanations for this [34]. First, it can be related to the different disease courses; in patients with asthma, the use of medication can, for example, be more dependent on the season than in patients with COPD [34]. Second, patients with COPD generally experience more consistent and severe disease symptoms [34]. Third, older age is associated with being more adherent, and patients with COPD are generally older than patients with asthma [35].

The findings of this study should be interpreted in light of several strengths and limitations. A major strength of this study pertains to the large amount of pharmacy dispensing data stemming from thousands of patients from hundreds of pharmacies geographically located throughout different areas in the Netherlands. This is likely to benefit the generalizability of the study results. In addition, these kinds of trials can contribute to external validity more than a randomized controlled trial [36]. Furthermore, the data set allowed for longitudinal research comparing data before and after the implementation of SARA with continuous enrollment of patients instead of during a specific period of time. For that reason, the impact of seasonal effects or national guidelines are expected to have been limited. Regarding the study limitations, the study results were based on retrospective pharmacy dispensing data. This design has several limitations, such as data that were not originally designed to answer specific research questions. Indeed, pharmacy dispensing data were limited in terms of not providing information about actual usage of the medication, more specifically if, when, and how often dispensed medication was used. Still, dispensing data are commonly used as a proxy for medication adherence [37,38]. Future studies could consider including other measures of medication adherence, for example, self-reports of medication use, smart inhaler devices, or measurements of metabolite levels [37,39-41]. Another study limitation is related to the commonly used “at least one” method to calculate the PDC as an indicator of medication adherence. This methodology does not take into account potential overuse of medication. Besides, the PDC can slightly differ when using the highest stock records of medication [42,43]. In addition, our assumption when interpreting the results was that better medication adherence was a consequence of better self-management skills. However, it could be the case that lower medication adherence is a sign of good self-management, as the patients may only take their medication when actually needed. This is an interesting topic for future research. In addition, future research could combine multiple methods to calculate medication adherence to provide a more comprehensive picture of this outcome measure. A recent publication by Menditto et al [43] proposes measuring persistence as a pragmatic and informative measure of medication adherence behaviors, which would allow for benchmarking of adherence strategies. Such strategies would thus facilitate cross-study comparisons and might help to identify a gold standard for calculating medication adherence [37,38,44]. This pragmatic trial only allowed for adherence measures based on pharmacy dispensing data. More specifically, the PDC is a preferred method of assessing medication adherence in case of treatment with multiple types of medications. An alternative metric such as the medication possession ratio (MPR) would be unable to cover multiple medication treatments since its numeration is the sum of days supplied in the period. In case of multiple medications, the MPR has to be averaged for each individual medication, leading to skewed results with possibilities of invalid ratios over 100%. So there are biases, such as not taking into account overuse and stockpiling, but using the PDC was a well-considered choice.

Another study limitation was that it was unknown what kind or intensity of support was offered by pharmacists. Hence, different pharmacists may have provided different types of support to patients. Even though this is inherent to tailored interventions, it would be worthwhile to investigate *what* type of support has the most beneficial effect. This also includes identifying *when*, *how*, and *how much* support should be offered. Addressing these questions can help to develop and strengthen evidence-based interventions [45]. A final study limitation that needs to be mentioned was the difference in demographic characteristics between the SARA and control participants. More specifically, SARA participants were generally younger and more often female. Even though such differences are not unusual in nonrandomized studies, they may have created selection bias [46]. However, SARA was, in principle, offered to all kinds of participants with varying degrees of symptoms. Therefore, the possibly biased selection of participants in the SARA group is likely to be representative of the group of potential future users of eHealth interventions for these groups. An important aspect to also take into account is that the questionnaire for the SARA intervention might increase patients’ awareness for medication adherence, but it is unlikely that this strongly affected adherence behavior directly. In future research, this could be something to take into account. More research is needed to draw firm conclusions on the effectiveness of SARA. A randomized controlled trial is needed to allow causal conclusions, which can then be used for a cost-effectiveness analysis as well, where, next to pharmacy dispensing data, other data can be collected, such as the following: (1) other sources that measure medication adherence, (2) objective data regarding exacerbation rates, (3) the actual and correct use of inhalation medication, and (4) health system characteristics that may impact adherence (eg, patient-provider interaction quality and procedural elements) [46]. In addition, qualitative research would allow for more insight into user experiences and could subsequently be used to optimize the intervention. In parallel, it would be interesting to investigate patients’ acceptability and effectiveness of the different components of the SARA intervention (eg, education materials and online support by a pharmacist). Also, it would be worthwhile to get a better understanding of the pharmacist perspective, for instance, what is their attitude toward eHealth in general and SARA specifically, what is the usability of SARA, and how is SARA used in the pharmacy (ie, does it add to the efficiency of care processes?)? Another recommendation for future research is to analyze the long-term effectiveness of SARA.

This research shows that SARA has the potential to help patients in decreasing exacerbation rates and improving medication adherence. Before large-scale implementation, it would be valuable to investigate both the patient and pharmacist perspective more thoroughly, both quantitatively and qualitatively. In this way, the full potential of the intervention can be maximized, making sure the intervention fits the needs and preferences of both of these stakeholders. Implementation barriers and facilitators can be investigated and taken into account when considering implementation strategies, such as integration of SARA into the workflow of pharmacists as well as the capacities of pharmacists to offer tailored follow-up care [47,48].

### Conclusions

This was the first study that assessed the effectiveness of a multi-component eHealth intervention stimulating correct use of medication. The results suggest that such an intervention has the potential to decrease exacerbation rates and improve medication adherence. This could subsequently have important clinical implications and lead to better patient outcomes and potentially reduced health care costs.

## Appendix

Multimedia Appendix 1 The 7-item questionnaire included in the SARA eHealth intervention. SARA: Service Apothecary Respiratory Advice; in Dutch, Service Apotheek Raad en Advies.

Multimedia Appendix 2 Results of exploratory analyses.

## Abbreviations

ATC: Anatomical Therapeutic Chemical
COPD: chronic obstructive pulmonary disease
COST: European Cooperation in Science and Technology
ICS: inhaled corticosteroids
MEC: Medical Ethics Committee
MPR: medication possession ratio
PDC: proportion of days covered
SARA: Service Apothecary Respiratory Advice; in Dutch, Service Apotheek Raad en Advies
WHO: World Health Organization
