# Supplementary material for: A Pharmacy-Based eHealth Intervention Promoting Correct Use of Medication in Patients With Asthma and COPD: Nonrandomized Pre-Post Study
Source: J Med Internet Res. 2022 Jun 8;24(6):e32396. doi: 10.2196/32396 (PMC9218880; doi:10.2196/32396)
Supplement: Multimedia Appendix 1 [file jmir_v24i6e32396_app1.docx]

Multimedia Appendix 1. Translated 7-item questionnaire of SARA

**Question 1.**

*Did you already start with [name medication]?*

Yes 1.1: Are you using [name medication] according to the prescribed dose**?**

Yes

No, I use less

No, I use more

No 1.2: Why did you not start with [name medication]?

My complaints are gone or have been reduced

I do not want to use this medication

I am afraid of the side effects

It is too expensive

Other [free space to fill in]

No, not yet picked up at the pharmacy 1.3: are you planning on using [name medication]?

**Question 2.**  (Asked if answer to question 1 is Yes)

*Do you like [name medication]?*

I am (very) satisfied about [name medication]

I am pretty satisfied about [name medication]

I am dissatisfied about [name medication]

I stopped

*2.1Why did you stop with [name medication] (multiple answers possible)*

My complains are gone or reduced

The medication did not work

I experienced side effects

I had problems with the use/intake of [name medication]

I forgot

Other [free space to fill in]

**Question 3.** (Asked if answer to question 2 is **not** I stopped)

*What do you expect about the effect of [name medication] (multiple answers possible)?*

Effectivity

Quality of life

Prognosis, healing, morbidity, mortality

I do not know what I can expect

Other [free space to fill in]

**Question 4.** *Did you experience problems when using [name medication] in the first weeks (multiple answers possible)?*

No

Yes [list of problems]

I experience side effects [space to fill in 5 side effects

I forget to take [name medication]

I am struggling with the time I need to take [name medication]

I have trouble opening the package

I find it hard to swallow [name medication]

I find it hard to inhale [name medication]

Other, [free space to fill in]

**Question 5.** *Are you worried about the use on the long term, and if so, what are you worries (multiple answers possible)?*

I am not worried

I am worried if [name medication] is effective enough

I am worried if [name medication] damages my body

I am worried about the side effects

I am wondering if I can take [name medication] with other medications

I am worried I do not use [name medication] the way it is meant to

Other [free space to fill in]

**Question 6*.*** *Do you have questions about the use, mechanisms or other things?*

No

Yes 🡪 fill in on which questions you would like an answer (multiple answers possible)

How does it work?

What are the side effects?

How long do I have to use this medication?

What is the best time to take [name medication]?

Can I take this medication with other medications?

Will it influence my driving behaviour/ Can I drive with [name medication]?

Is it reimbursed by my health insurer?

**Question 7.** *Do you want an appointment to discuss your questions/worries?*

Yes

No
