# Supplementary material for: A Pharmacy-Based eHealth Intervention Promoting Correct Use of Medication in Patients With Asthma and COPD: Nonrandomized Pre-Post Study
Source: J Med Internet Res. 2022 Jun 8;24(6):e32396. doi: 10.2196/32396 (PMC9218880; doi:10.2196/32396)
Supplement: Multimedia Appendix 2 [file jmir_v24i6e32396_app2.docx]

Multimedia Appendix 2: Results of exploratory analyses

**Table S1. Demographic characteristics of the study population(s) analyzed for the different outcome measures. Data are provided as means (*SD*) or as counts (percentages).**

|  |  |  | **Study group** | | **Total population** |
| --- | --- | --- | --- | --- | --- |
|  |  |  | **SARA** | **Control** |  |
| **Outcome measure: Exacerbation rate** |  |  |  |  |  |
| - Asthma |  |  | *n =* 1459 | *n =* 3921 | *n =* 5380 |
|  | Gender* | Male | 463 (31.7%) | 1485 (37.9%) | 1948 (36.2%) |
|  |  | Female | 987 (67.6%) | 2416 (61.6%) | 3403 (63.3%) |
|  |  | Unknown | 9 (0.6%) | 20 (0.5%) | 29 (0.5%) |
|  | Age* |  | 54.1 (14.7) | 58.0 (16.4) | 56.9 (16.0) |
| - COPD |  |  | *n =* 941 | *n =* 3131 | *n =* 4072 |
|  | Gender | Male | 419 (44.5%) | 1366 (43.6%) | 1785 (43.8%) |
|  |  | Female | 517 (4.9%) | 1757 (56.1%) | 2274 (55.8%) |
|  |  | Unknown | 5 (0.5%) | 8 (0.3%) | 13 (0.3%) |
|  | Age* |  | 63.3 (10.0) | 66.8 (12.1) | 66.0 (11.7) |
| **Outcome measure: Medication adherence** | | | | |  |
| Subpopulation: New users^a^ | | | | |  |
| - Asthma |  |  | *n* = 233 | *n* = 649 | *n* = 882 |
|  | Gender | Male | 81 (34.8%) | 230 (35.4%) | 311 (35.3%) |
|  |  | Female | 152 (65.2%) | 415 (63.9%) | 567 (64.3%) |
|  |  | Unknown | 0 | 4 (0.6%) | 4 (0.5%) |
|  | Age* |  | 56.2 (15.0) | 58.6 (17.3) | 58.0 (16.8) |
| - COPD |  |  | *n* = 121 | *n* = 435 | *n* = 556 |
|  | Gender | Male | 47 (38.8%) | 190 (43.7%) | 237 (42.6%) |
|  |  | Female | 73 (60.3%) | 243 (55.9%) | 316 (56.8%) |
|  |  | Unknown | 1 (0.8%) | 2 (0.5%) | 3 (0.5%) |
|  | Age* |  | 65.6 (9.9) | 68.7 (12.9) | 68.0 (12.4) |
| Subpopulation:Chronic users^b^ | | | | | |
| - Asthma |  |  | *n* = 849 | *n* = 2266 | *n* = 3115 |
|  | Gender* | Male | 263 (31.0%) | 876 (38.7%) | 1139 (36.6) |
|  |  | Female | 579 (68.2%) | 1378 (60.8%) | 1957 (62.8) |
|  |  | Unknown | 7 (0.8%) | 12 (0.5%) | 19 (0.6%) |
|  | Age* |  | 57.6 (14.1) | 69.4 (11.4) | 61.0 (15.0) |
| - COPD |  |  | *n* = 676 | *n* = 2110 | *n* = 2786 |
|  | Gender | Male | 302 (44.7%) | 904 (42.8%) | 1206 (43.4%) |
|  |  | Female | 371 (54.9%) | 1203 (57.0%) | 1574 (56.5%) |
|  |  | Unknown | 3 (0.4%) | 3 (0.1%) | 6 (0.2%) |
|  | Age* |  | 65.9 (10.1) | 62.3 (15.2) | 68.6 (11.2) |
| **Outcome measure: Antimycotic treatment** | | | | |  |
| - Asthma |  |  | *n =* 440 | *n =* 1046 | *n =* 1486 |
|  | Gender* | Male | 118 (26.8%) | 366 (35.0%) | 484 (32.6%) |
|  |  | Female | 320 (72.7%) | 675 (64.5%) | 995 (67%) |
|  |  | Unknown | 2 (0.5%) | 5 (0.5%) | 7 (0.5) |
|  | Age* |  | 52.3 (14.8) | 55.4 (16.9) | 54.47 (16.4) |
| - COPD |  |  | *n =* 186 | *n =* 661 | *n =* 847 |
|  | Gender | Male | 78 (41.9%) | 246 (37.2%) | 324 (38.3%) |
|  |  | Female | 108 (58.1%) | 415 (62.8%) | 523 (61.7%) |
|  |  | Unknown | 0 | 0 | 0 |
|  | Age* |  | 61.6 (10.1) | 64.8 (13.0) | 64.14 (12.5) |

*Note:* COPD = Chronic Obstructive Pulmonary Disease; SARA = eHealth intervention Service Pharmacy Advice (in Dutch ‘Service Apotheek Raad en Advies’)

^a^ Participants with zero R03-dispensing records in the year before the index date

^b^ Participants having ≥ 1 R03-dispensing records in the year before the index date

*Significant difference between the SARA and the control condition (*p*<0.05)

**Table S2. Data of the outcome measure exacerbation rates displayed per disease indication**

| **Descriptives** | | | | | | |  | | **Statistics** | | | |
| --- | --- | --- | --- | --- | --- | --- | --- | --- | --- | --- | --- | --- |
| **Study subpopulation** | **Period^a^** | **Study group** | **Exacerbation rates**  ***M (SD)*** | **Difference score^b^** | ***N*** |  | | **t(df)** | | ***P*-value** | **95% CI** | **Cohen *d*** |
| Asthma |  |  |  |  |  |  | | 2.97(2820) | | .003 | 0.036 – 0.177 | 0.11 |
|  | Year before | Control | 0.55 (1.0) |  | 3921 |  | |  | |  |  |  |
|  | Year after | Control | 0.72 (1.1) | 0.17 | 3921 |  | |  | |  |  |  |
|  | Year before | SARA | 0.54 (1.0) |  | 1459 |  | |  | |  |  |  |
|  | Year after | SARA | 0.61 (1.0) | 0.07 | 1459 |  | |  | |  |  |  |
| COPD |  |  |  |  |  |  | | 1.67 (4070) | | .09 | -0.016 – 0.207 | 0.05 |
|  | Year before | Control | 0.82 (1.3) |  | 3131 |  | |  | |  |  |  |
|  | Year after | Control | 0.94 (1.5) | 0.12 | 3131 |  | |  | |  |  |  |
|  | Year before | SARA | 0.88 (1.4) |  | 941 |  | |  | |  |  |  |
|  | Year after | SARA | 0.91 (1.4) | 0.03 | 941 |  | |  | |  |  |  |

*Note:* CI = Confidence Interval; COPD = Chronic Obstructive Pulmonary Disease; M = Mean SARA= Intervention ‘Service Pharmacy Advice’ (in Dutch ‘*Service Apotheek Raad en Advies’)*; SD = Standard Deviation

^a^ One year before or one year after the implementation of SARA

^b^ Difference score of the year after SARA minus the year before SARA

**Table S3. Data of the outcome measure medication adherence displayed per disease indication and subpopulation**

| **Descriptives** | | | | | | |  |  | **Statistics** | | | |
| --- | --- | --- | --- | --- | --- | --- | --- | --- | --- | --- | --- | --- |
| **Study subpopulation** | **Period^a^** | **Study group** | **PDC**  ***M (SD)*** | **Days covered**  ***M (SD)*** | **Difference score^b^** | ***N*** |  |  | ***t(df)*** | ***P*-value** | **95% CI** | **Cohen *d*** |
| Chronic users and asthma |  |  |  |  |  |  |  |  | -1.86 (1500) | .06 | -4.148 – 0.114 | -0.10 |
|  | Year before | Control | 70.94 (28.6) | 258.93 (104.3) |  | 2261 |  |  |  |  |  |  |
|  | Year after | Control | 76.76 (24.6) | 280.18 (89.8) | 5.82 | 2261 |  |  |  |  |  |  |
|  | Year before | SARA | 66.92 (30.5) | 244.24 (111.5) |  | 845 |  |  |  |  |  |  |
|  | Year after | SARA | 74.76 (25.5) | 272.86 (93.1) | 7.84 | 845 |  |  |  |  |  |  |
|  |  |  |  |  |  |  |  |  |  |  |  |  |
| Male: Chronic users and COPD |  |  |  |  |  |  |  |  | -2.80 (1201) | .005 | -9.391 - -1.654 | -0.16 |
|  | Year before | Control | 76.32 (27.8) | 278.58 (101.4) |  | 901 |  |  |  |  |  |  |
|  | Year after | Control | 77.96 (27.2) | 284.58 (99.3) | 1.64 | 901 |  |  |  |  |  |  |
|  | Year before | SARA | 74.82 (29.2) | 273.11 (106.4) |  | 302 |  |  |  |  |  |  |
|  | Year after | SARA | 82.98 (22.8) | 299.25 (83.2) | 8.16 | 302 |  |  |  |  |  |  |
| Female: Chronic users and COPD |  |  |  |  |  |  |  |  | 0.13(1571) | 0.9 | -2.957 – 3.394 | 0.01 |
|  | Year before | Control | 75.49 (27.9) | 275.52 (101.7) |  | 1203 |  |  |  |  |  |  |
|  | Year after | Control | 79.59 (24.5) | 290.50 (89.5) | 4.10 | 1203 |  |  |  |  |  |  |
|  | Year before | SARA | 75.16 (27.4) | 274.35 (100.1) |  | 370 |  |  |  |  |  |  |
|  | Year before | SARA | 79.05 (24.8) | 288.52 (90.5) | 3.89 | 370 |  |  |  |  |  |  |

*Note:* CI = Confidence Interval; COPD = Chronic Obstructive Pulmonary Disease; SARA= Intervention ‘Service Pharmacy Advice’ (in Dutch ‘*Service Apotheek Raad en Advies’)*; PDC = proportion of days covered; df= degrees of freedom; M = Mean; SD = Standard Deviation;

^a^ One year before or one year after the implementation of SARA

^b^ Difference score of the year after SARA minus the year before SARA

**Table S4. Exploratory results of the type of user effect in terms of medication adherence rates one year after the implementation of SARA.**

| **Descriptives** | | | | | |  | **Statistics** | | | |
| --- | --- | --- | --- | --- | --- | --- | --- | --- | --- | --- |
| **Study subpopulation** | **Period^a^** | **Study group** | **PDC**  ***M (SD)*** | **Days covered**  ***M (SD)*** | ***N*** |  | ***t(df)*** | ***P*-value** | **95% CI** | **Cohen *d*** |
| **New users total** |  |  |  |  |  |  | -1.85 (1434) | .06 | -5.604 – 0.160 | -0.10 |
|  | Year after | SARA | 66.17 (23.1) | 241.52 (84.3) | 353 |  |  |  |  |  |
|  | Year after | Control | 63.45 (24.2) | 231.48 (88.5) | 1083 |  |  |  |  |  |
| **New users asthma** |  |  |  |  |  |  | -0.90 (878) | .37 | -5.302 – 1.971 | -0.06 |
|  | Year after | SARA | 63.70 (23.4) | 232.52 (85.4) | 232 |  |  |  |  |  |
|  | Year after | Control | 62.04 (24.5) | 226.44 (89.4) | 648 |  |  |  |  |  |
| **New users COPD** |  |  |  |  |  |  | -2.34 (206) | .02 | -9.860 - -0.839 | -0.33 |
|  | Year after | SARA | 70.89 (21.8) | 258.76 (79.7) | 121 |  |  |  |  |  |
|  | Year after | Control | 65.54 (23.7) | 239.23 (86.7) | 435 |  |  |  |  |  |

*Note:* CI = Confidence Interval; COPD = Chronic Obstructive Pulmonary Disease; SARA= eHealth intervention Service Pharmacy Advice (in Dutch ‘*Service Apotheek Raad en Advies’)*; PDC = proportion of days covered; df= degrees of freedom; M = Mean; SD = Standard Deviation;

^a^ one year after the implementation of SARA

**Table S5. Data of the outcome measure use of antimycotics displayed per disease indication**

| **Descriptives** | | | | | **Statistics** | | | | | |
| --- | --- | --- | --- | --- | --- | --- | --- | --- | --- | --- |
| **Study subpopulation** | **Period^a^** | **Study group** | **Prescribed**  **antimycotics (%)** | ***N*** |  | ***t(df)*** | ***P*-value** | **95% CI** | **Cohen *d*** |  |
| **Asthma** |  |  |  |  |  | 0.35(2968) | 0.73 | -0.519 – 0.743 | 0.01 |  |
|  | Year before | Control | 4.9 | 1046 |  |  |  |  |  |  |
|  | Year after | Control | 5.7 | 1046 |  |  |  |  |  |  |
|  | Year before | SARA | 6.1 | 440 |  |  |  |  |  |  |
|  | Year after | SARA | 6.1 | 440 |  |  |  |  |  |  |
| **COPD** |  |  |  |  |  | 0.49(1690) | 0.79 | -1.084 – 0.831 | 0.02 |  |
|  | Year before | Control | 4.4 | 661 |  |  |  |  |  |  |
|  | Year after | Control | 6.7 | 661 |  |  |  |  |  |  |
|  | Year before | SARA | 3.8 | 186 |  |  |  |  |  |  |
|  | Year after | SARA | 7.0 | 186 |  |  |  |  |  |  |

*Note*: CI = Confidence Interval; COPD = Chronic Obstructive Pulmonary Disease; SARA= Intervention ‘Service Pharmacy Advice’ (in Dutch ‘*Service Apotheek Raad en Advies’)*; df= degrees of freedom; M = Mean; SD = Standard Deviation;

^a^ One year before or one year after the implementation of SARA
